# HHI-AttentionNet: An Enhanced Human-Human Interaction Recognition Method Based on a Lightweight Deep Learning Model with Attention Network from CSI

**DOI:** 10.3390/s22166018

**Published:** 2022-08-12

**Authors:** Islam Md Shafiqul, Mir Kanon Ara Jannat, Jin-Woo Kim, Soo-Wook Lee, Sung-Hyun Yang

**Affiliations:** 1 Department of Electronic Engineering, Kwangwoon University, Seoul 01897, Korea; 2 Kwangwoon Academy, Kwangwoon University, Seoul 01897, Korea

**Keywords:** human activity recognition (HAR), human-human interactions (HHIs), channel state information (CSI), deep learning (DL), antenna-frame-subcarrier attention mechanism (AFSAM)

## Abstract

Nowadays WiFi based human activity recognition (WiFi-HAR) has gained much attraction in an indoor environment due to its various benefits, including privacy and security, device free sensing, and cost-effectiveness. Recognition of human-human interactions (HHIs) using channel state information (CSI) signals is still challenging. Although some deep learning (DL) based architectures have been proposed in this regard, most of them suffer from limited recognition accuracy and are unable to support low computation resource devices due to having a large number of model parameters. To address these issues, we propose a dynamic method using a lightweight DL model (HHI-AttentionNet) to automatically recognize HHIs, which significantly reduces the parameters with increased recognition accuracy. In addition, we present an Antenna-Frame-Subcarrier Attention Mechanism (AFSAM) in our model that enhances the representational capability to recognize HHIs correctly. As a result, the HHI-AttentionNet model focuses on the most significant features, ignoring the irrelevant features, and reduces the impact of the complexity on the CSI signal. We evaluated the performance of the proposed HHI-AttentionNet model on a publicly available CSI-based HHI dataset collected from 40 individual pairs of subjects who performed 13 different HHIs. Its performance is also compared with other existing methods. These proved that the HHI-AttentionNet is the best model providing an average accuracy, F1 score, Cohen’s Kappa, and Matthews correlation coefficient of 95.47%, 95.45%, 0.951%, and 0.950%, respectively, for recognition of 13 HHIs. It outperforms the best existing model’s accuracy by more than 4%.

## 1. Introduction

Human activity recognition (HAR) aims to determine the current behaviors and intentions of human movement based on a sequence of observations made regarding human activities and their surrounding circumstances using Artificial Intelligence (AI). HAR is currently a vital and popular research area due to its numerous applications in various fields such as health monitoring, analysis of sports events [1], entertainment events [2], home care for the aging person [3], etc. The literature reveals [4,5,6] that computer vision and inertial sensor-based techniques are commonly employed for HAR. However, both of these methods have their own limitations. Computer vision-based HAR methods are high cost due to expensive cameras, privacy violations, object occlusion, etc. [7]. Furthermore, the camera needs to be set up in advance, and its performance is affected by the ambient lighting; humans also need to be within the camera’s visual range, and it is unable to distinguish actions when there are walls or other impediments present. The main problem with wearable inertial sensors are user inconvenience, obtrusiveness, and maintenance costs. Wearable or inertial sensor-based techniques always force the users to wear a variety of tracking devices, which are bothersome and inconvenient for the user [7].

WiFi-HAR methods [4,8] have emerged as a solution because of their ability to overcome the aforementioned limitations. Advantages include (i) low cost, (ii) no privacy violation, (iii) compact size, (iv) contactless, and (v) hardware facilities are universal. In addition, with the widespread installation of commodity WiFi devices in homes, HAR methods based on WiFi have attracted more interest. Though WiFi-HAR has tremendous advantages in an indoor environment, it has several drawbacks such as short range of coverage and limitations in the case of multi-user scenarios. In a WiFi-HAR system, received signal strength indicator (RSSI), specialized radio hardware-based signals, and channel state information (CSI) are the three types of WiFi signals used to detect human activity. The RSSI signal has been employed in various sensing applications, including indoor location [9], tracking [10], and radio tomographic imaging (RTM) [11]. However, it is difficult to achieve high accuracy on fine-grained HAR from RSSI signals because of its limited range accuracy, inconsistent readings, and low resolution. Furthermore, the specialized radio hardware is not a commercially available product and as a result, it is more costly to set up.

CSI contains information on how WiFi signals are propagated between the transmitting and receiving antenna at a particular carrier frequency. CSI works with Orthogonal Frequency-Division Multiplexing based on multiple input multiple output schemes that provide more information about the phase and amplitude of each sub-carrier [12]. The primary idea behind HAR through the CSI signal is that when things or humans move between the transmitting and receiving antennas, the moving body affects the multipath propagation. Various moves have different consequences depending on how the body moves between the antennas. CSI can easily detect the information of different movements in the surroundings. In addition, the literature reveals that CSI-based HAR shows considerably better performance than RSSI [13]. This is because CSI is a fine-grained signal and the phase and amplitude of the CSI signal easily differentiate static and non-static objects between transmitter and receiver. Researchers have used WiFi based CSI signals for several applications, such as detecting micro-movement to hear words [14], gesture recognition [15], user identification and localization [16], driver activity recognition [17], handwriting recognition [18], pose estimation [19], and fall detection [20].

DL-based models such as convolutional neural networks (CNNs) and long short-term memory (LSTM) have been shown to perform better than the traditional feature-based classifiers for HAR from CSI signals (e.g., [4,13,21] vs. [22,23,24,25]). Despite the amazing results that have been obtained with the current CSI-based human activity identification systems, their main focus has been on identifying single human activities (SHA) that are performed by a single person [4,13,26]. Because of this, the applicability of these methods may be limited in situations that occur in the real world and involve multiple individuals. In this regard, previous studies [27,28] have indicated that detecting/recognizing human-human interactions (HHIs), in which two people interact with one another (for example, handshakes and hugs), is considered more challenging than recognizing SHA (e.g., running and standing activities) due to the following reasons. First, HHIs are based on the interdependencies and causal linkages between the moving body parts of the two individuals involved. Second, HHIs include a wide range of differences between individuals and how interaction are performed between them. Third, distinct HHIs may entail similar movements by the two interacting humans.

In this study, we proposed a lightweight deep learning model (HHI-AttentionNet) to automatically recognize HHIs and reduce model parameters without sacrificing recognition accuracy. The HHI-AttentionNet composed of a depthwise separable convolution (DS-Conv) block for feature extraction and added antenna-frame-subcarrier attention mechanism (AFSAM) to focus on the most significant features, aims to reduce the impact of the complexity on the CSI signal as well as to improve the model’s capability to recognize HHIs. Thus the main contributions of the paper are as follows:

- A lightweight DL model (HHI-AttentionNet) has been proposed to improve the recognition accuracy of HHIs;
- An AFSAM that combines the antenna attention module (*AAM*) and frame-subcarrier attention module (*FSAM*) is designed in the HHI-AttentionNet model to improve the representative capability of the proposed model for recognizing HHIs correctly;
- A comparative study of different methods for HHI recognition and comparison of their performance;
- The proposed method could be the best-suited sophisticated method for recognizing both HHIs and single human activity because of its high-level activity recognition ability with a limited number of parameters.

## 2. Related Work

WiFi based human activity recognition (WiFi-HAR) has recently gained immense attention in an indoor environment among the existing techniques due to its tremendous advantages, including ubiquitous availability, non-light of sight communication and contactless sensing, etc. Current research on human activity recognition (HAR) using WiFi can be classified into RSSI-based and CSI-based methods.

### 2.1. RSSI-Based Methods

RSSI-based HAR approaches utilize the power of signal changes caused by human activities [23]. The RSSI measures the variance in received signal strength over time. The authors [29] proposed a device-free system for detecting human activity in indoor circumstances. They collected RSSI data from multiple mobile phones through multiple access points and stored data to train different ML models. They used five ML models to validate their data and achieved 95% accuracy in real-time. Sigg et al. [30] proposed a passive and device-free HAR system based on RSSI signals obtained from mobile phones. They extracted 18 different features and selected only 9 features using feature selection. Those selected features were then fed to the k-nearest neighbor (KNN) algorithm and achieved 52% accuracy when detecting 11 gestures and 72% accuracy when detecting 4 gestures. Jing et al. [31] designed a low-cost HAR system based on an RSSI coarse-to-fine hierarchical DL framework. They used the ESP8266 sensor to reduce the installation cost and collect RSSI data from two scenarios: an empty room and a bedroom. They used SVM and gated recurrent unit (GRU) to validate their dataset and claimed better results from GRU than the traditional methods. Wang et al. [32] extracted the wavelet feature from RSSI to build a HAR system. They showed that wavelet features can provide reliable identification features for HAR and generate high performance of the proposed system. The experiments’ findings demonstrated that the accuracy level was greater than 90%. Huang et al. [33] designed a deep CNN to detect a person using a WiFi-based RSSI signal. They mixed the raw RSSI values with the wavelet coefficients as the CNN’s input to differentiate changes in the signal induced by human movement. Their proposed system recognised walking behavior with a 95.5% accuracy rate. To accurately characterize RSSI measurements, Gu et al. [34] proposed a fusion technique based on a classification tree to detect human activity. Their proposed method achieved an average accuracy of 72.47%. RSSI is mainly used in short-distance ranging and indoor positioning. However, the RSSI signal does not work well when the signal is variegated and in a complex environment.

### 2.2. CSI-Based Methods

Recently, CSI has been utilized for indoor localization and classification of human activity as compared to RSSI because it offers a finer-grained representation of the wireless link. Wang et al. [19] proposed a system to detect human activity and indoor localization. They developed a dataset for six distinct activities and designed a multi-task 1D CNN where basic architecture is based on ResNet. The proposed architecture attained an accuracy of 88.13% and 95.68% on average for activity recognition and indoor localization, respectively. Yang et al. [35] created a framework for HAR using a WiFi CSI signal with three modules. Firstly, they proposed an antenna selection algorithm that automatically chose the antenna based on its sensitivity to different activities. After that, they presented two signal enhancement algorithms to improve active signals besides weakening inactive ones. Finally, they proposed a segmentation algorithm to find an activity’s starting and finishing point. Damodaran et al. [36] presented a HAR system that can classify five classes from the CSI signal. They collected data from two scenarios: a Line of Sight (LOS) and a Non-Line of Sight (N-LOS) scenario in an indoor environment. They evaluated the performance of two different algorithms, SVM and LSTM, on the same data set and observed that LSTM requires less preprocessing and achieved 97.33% average accuracy on the LOS scenario. Yousefi et al. [37] developed a dataset for HAR from WiFi named StanWiFi, which contains seven different activities. They extracted different statistical features and employed three different models (hidden Markov model, LSTM, and a random forest) to classify the activities and reported an average accuracy of 64.6%, 73.3%, and 90.5%, respectively. Heju et al. [8] proposed an indoor HAR system based on a WiFi signal named Wi-motion. They extracted features from both amplitude and phase. They used a posterior probability vector-based strategy rather than a single classifier and reported an average accuracy of 96.6% in LOS scenarios. Santosh et al. [13] proposed a modified Inception Time network architecture called CSITime for HAR based on WiFi CSI signal. They used three datasets, namely ARIL, StanWiFi, and SignFi datasets, to evaluate their system and achieved an accuracy of 98.20%, 98%, and 95.42% respectively. A CSI-based CARM theory was introduced by Wang et al. [38] based on two methodologies: the CSI-speed model and the CSI-activity model. They claim that the CARM is resistant to environmental changes and has a recognition accuracy of 96%. Huan et al. [39] presented a CSI-based HAR system that used the relationship between body movement and amplitude to identify different activities. They developed an Adaptive Activity Cutting Algorithm (AACA) and gained an average accuracy of 94.20%. Muaaz et al. [40] proposed an environment-independent approach to recognize four different human activities. They generated spectrogram images using STFT as an input of CNN and achieved a 97.78% result. Alazrai et al. [41] proposed an end-to-end DL framework named E2EDLF consisting of three-block CNN. They converted the raw signal into two-dimensional images and then fed those images to E2EDLF to classify HHIs. They achieved an accuracy of 86.3%. Kabir et al. [42] developed a deep-learning-based CSI-IANet for recognizing HHIs. As the conversion of CSI signal to gray-scale image reduces the available features, so they directly fed CSI signals to recognize HHIs after denoising. They also claimed an average accuracy of 91.30% and an F1 score of 93.0% with high computational complexity. 

From the above discussion, we can see that most of the researchers have worked on single user HAR and achieved sufficient accuracy, whereas very few works have been done with multi-user HHI recognition. Multi-user HHI recognition has suffered from low recognition accuracy, the number of parameters, and recognition time. However, we proposed a lightweight DL model comprised of the depthwise separable convolution (DS-Conv) and attention mechanism to recognize HHIs. Therefore, our model showed better performance for recognizing HHIs in terms of accuracy, number of parameters, and recognition time than the existing solutions.

## 3. Dataset

In our work, we have used a publicly available CSI-based HHI [43] dataset to evaluate the performance of our proposed model. This dataset has 12 different interactions. The dataset includes 40 individual pairs made from 66 healthy people who voluntarily agreed to participate in this experiment. Each of the 40 pairs was told to do ten different trials of the 12 distinct HHIs in an indoor position. The total number of trials recorded on their dataset stands at 4800. Each of the 12 interactions consists of two intervals, one being the steady-state and the other being the interaction interval. The two participants stand in front of each other without doing any action at a steady state. On the other hand, each pair takes part in one of 12 different HHI actions during the interaction period. As a result, the CSI dataset has thirteen HHIs classes, including the steady-state interaction and the twelve HHIs. They used Sagemcom 2704 as an access point and a desktop computer provided with an Intel 5300 NIC as a receiver. The WiFi signals were recorded using the online Linux 802.11n CSI tool [44]. The access point was set up to run at 2.4 GHz with wireless channel number 6, a channel bandwidth of 20 MHz, and an index eight modulation coding scheme. The NIC has three external receiver antennas (Nrx = 3), while the access point has two internal transmission antennas (Ntx = 2). Thus, the system comprises 2 × 3 WiFi streams. The CSI tool can capture the CSI for 30 subcarriers (i.e., Nsc = 30). Therefore, for the MIMO-OFDM system, each packet contains 180 CSI values. The overall dataset statistics are given in Table 1. 

## 4. Background of CSI

CSI contains the channel properties of any wireless communication system. In the communication system, when a transmitting signal comes into contact with an obstacle like a wall, furniture, ceiling, or person, it is scattered, deflected, and reflected before going to the receiver. CSI can describe how a signal changes (i.e., time delay, amplitude attenuation, and phase shift) between the transmitter and receiver [20]. Wireless technology communication systems are advancing with adoption of Multiple Input Multiple Output (MIMO), consisting of multiple pairs of transmitting-receiving antennas. A MIMO channel’s available bandwidth is divided by the Orthogonal Frequency Division Multiplexing (OFDM) into several orthogonal subcarrier frequencies that are simultaneously transmitted. In particular, the following mathematical statements can be used to characterize the Multiple Input Multiple Output-Orthogonal Frequency-Division Multiplexing (MIMO-OFDM) communication system [8,20]:

(1) $$y_{i}=H_{i}x_{i}+v,\;i=1,\;2,\;3,\;\ldots ,\;N$$

where *H_i_* represents the complex matrix of the *i*th OFDM subcarrier, *v* represents noise, *N* represents the number of OFDM subcarriers. $y_{i}\in \;{\mathbb{R}}^{N_{R_{a}}}$ and $x_{i}\in \;{\mathbb{R}}^{N_{T_{a}}}$ are the transmitted and received signal where $N_{T_{a}}$ and $N_{R_{a}}\;$ denotes the number of transmitting and receiving antennas. The basic structure of *H_i_* is given bellow

(2) $$H_{i}=\left[\begin{array}{ccc} h_{i}^{T_{\;a_{1}}R_{a_{1}}} & \cdots & h_{i}^{T_{\;a_{j}}R_{a_{1}}}\\ \vdots & \ddots & \vdots \\ h_{i}^{T_{\;a_{j}}R_{a_{1}}} & \cdots & h_{i}^{T_{\;a_{j}}R_{a_{k}}} \end{array}\right]$$

Here,$\;h_{i}^{T_{\;a_{j}}R_{a_{k}}}$ represents the complex matrix of CSI of $i^{th}$ OFDM subcarrier between $j^{th}$ transmitted antenna and $k^{th}$ receiving antenna. $h_{i}^{T_{\;a_{j}}R_{a_{k}}}$ can be expressed as:

$$h_{i}^{T_{\;a_{j}}R_{a_{k}}}=|\;h_{i}^{T_{\;a_{j}}R_{a_{k}}}|\;e{∠}^{h_{i}^{T_{\;a_{j}}R_{a_{k}}}}$$

where |$h_{i}^{T_{\;a_{j}}R_{a_{k}}}$| and ${∠}^{h_{i}^{T_{\;a_{j}}R_{a_{k}}}}$ represents amplitude and phase value of CSI, respectively.

Although CSI contains amplitude and phase information, amplitude information is more stable than phase information [44] (where the carrier frequency offset (CFO) introduces unpredictable phase problems over several packets [38]). Hence, in this study, we consider only amplitude information of CSI to classify HHIs.

## 5. Proposed Methodology

**Figure 1:**
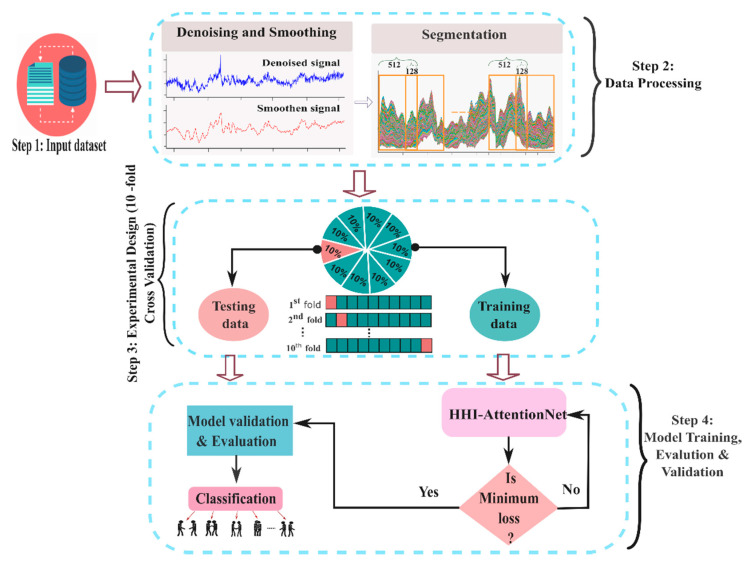
Block diagram of methodological steps to recognize HHI.

The block diagram of the proposed HHI-AttentionNet model is depicted in Figure 1. It contains a summary of the main steps involved in the recognition of HHIs. It is divided into four major parts: i. Load dataset; ii. Preprocessing of the raw CSI data; iii. Splitting of datasets into 10 fold; iv. HHI-AttentionNet model training, validation and evaluation.

### 5.1. Data Preprocessing

The data preprocessing section consists of two parts: (i) signal filtering and (ii) segmentation. The CSI-based HHI dataset [43] has a four-dimensional (4D) tensor, including the time-domain (i.e., packet index), frequency-domain (i.e., OFDM subcarrier frequencies), and spatial domain in the CRF values that are found for a WiFi system (i.e., pairs of transmitting-receiving antennas). The raw WiFi CSI data must be preprocessed before feeding any classifier or model because it contains high-frequency noise, outliers, and artifacts [23]. We used a Butterworth bandpass filter for denoising to remove noises from the CSI data. A bandpass filter is formed by merging a high-pass and low-pass filter. The low-pass and high-pass Butterworth filter is defined by Equations (3) and (4):

(3) |Hlp(jω)|≜11+ωωo2n 

(4) |Hhp(jω)|≜11+ωωo−2n 

where $\omega_{o}$ is the cut-off frequency in angular form, and n is the order of the filter.

To smooth the filtered signal, we used a Gaussian smoothing function which helps to suppress the short peaks; it is defined by Equation (5):

(5) $$g\left(x\right)=\frac{1}{\sqrt{2\pi \sigma }}e^{-\frac{x^{2}}{2\sigma^{2}}}\;$$

where σ is the standard deviation of the distribution.

**Figure 2:**
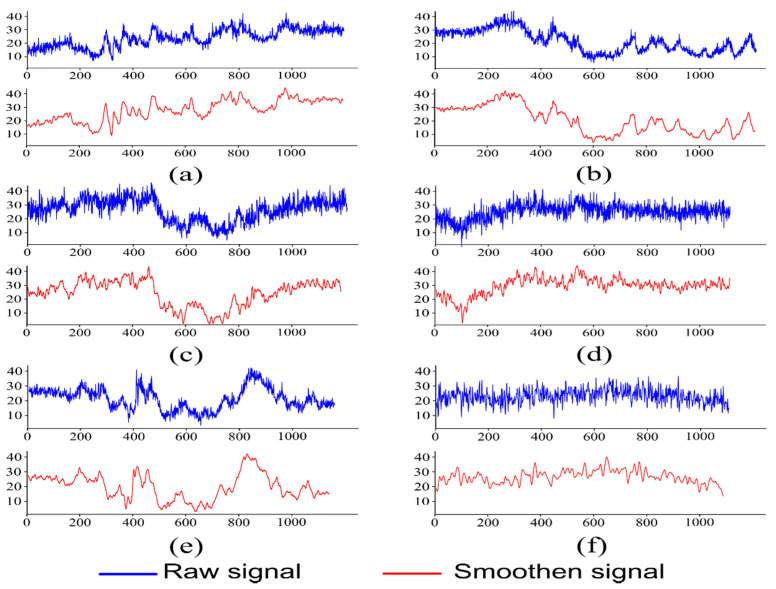
Raw and smoothing CSI signal visualization of some interactions, i.e., (**a**) Approaching, (**b**) Departing, (**c**) Handshaking, (**d**) High five, (**e**) Hugging, (**f**) Steady state.

The raw and denoising CSI signals of some interaction of the first subcarrier out of 30 subcarriers for the first transmitting and receiving antenna pairs are displayed in Figure 2. Following the process of denoising, the filtered CSI data in four dimensions are transformed into a two-dimensional matrix with the shape, *S* = *M* × *N* where, *M* = $N_{R_{a}}\times$ $N_{T_{a}}$ and *N* = number of OFDM subcarriers.

Segmentation: Segmentation is the way of splitting a signal into smaller parts or windows. We perform segmentation in our study for two reasons. The first reason is that the recorded signals are different subjects and their lengths are different; which limits the recognition process. Another issue is that processing a large length of data takes more time and requires more computing power. Therefore, a fixed-size window is used to split the processed CSI signal into several small signals. Every small signal is treated as an individual instance to train the HHI-AttentionNet model. Instances are generated from each record by selecting a window size of 512 and a stride of 128 (25% of 512 with an overlap of 75%).

### 5.2. HHI-AttentionNet

**Figure 3:**
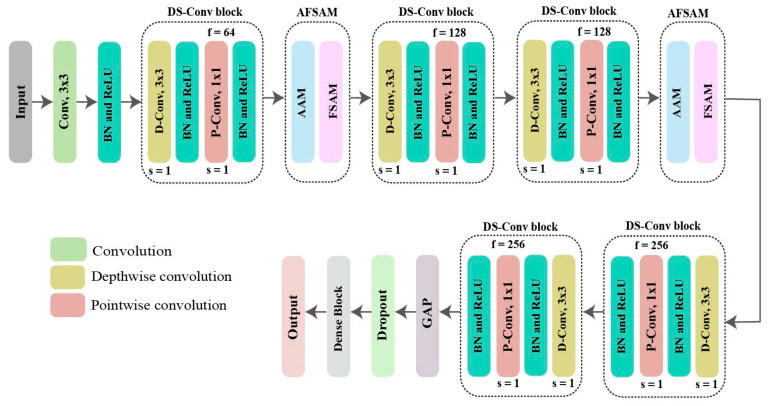
The architecture of our proposed model (HHI-AttentionNet).

Although several DL-based architectures have been proposed and achieved high performance in many fields, most of them require many parameters during their evaluation phase which does not fully satisfy the requirements of modern low-resource devices. To avoid this, we have utilized a convolutional neural network (CNN) algorithm where *DS-Conv* is implemented to reduce the number of parameters. Nowadays, some researchers have shown that using attention mechanisms improves CNNs’ overall performance. Motivated by them, we also proposed AFSAM, which is able to progressively determine the information that ought to be stressed or repressed, as well as identify the significance of various portions within the feature maps. As a result, our proposed HHI-AttentionNet model synergistically integrates *DS-Conv* and AFSAM to learn powerful feature representations while significantly reducing the number of parameters without sacrificing the accuracy of HHI recognition. Figure 3 shows the architecture of the HHI-AttentionNet, and a brief description of our proposed model is given below:

#### 5.2.1. Depthwise Separable Convolutional Block and Dense Block

We have designed two blocks: the depthwise separable convolution (*DS-Conv*) block and the dense block. Each block comprises of several layers. *DS-Conv* [45] is a factorized form of the standard or classic convolution (*S-CNN*). *S-CNN* combines both filter and input in one step to set output, whereas *DS-Conv* splits the whole *S-CNN* procedure into two parts. First, it learns the spatial domain utilizing depthwise convolution (*D-Conv*). Second, it combines the outputs of the *D-Conv*, called pointwise convolution (*P-Conv*).

Consider, a *S-CNN* taken as input *I_H_* × *I_W_* × *M* and that produces an output as *O_H_* × *O_W_* × *N*, where *I_H_*, *I_W_*, and *O_H_*, *O_W_* indicate the height and width of the input and output data, and *M*, *N* represent the input and output channel or depth. Any *S-CNN* layer is parameterized by the kernel or filter *K* of shape *K_H_* × *K_W_* × *M* × *N*, where *K_H_*, *K_W_* indicates the size of kernel or filter height and width. The following mathematical equation expresses the output and computational cost for any *S-CNN*:

(6) $$O{\left(S-CNN\right)}_{k,l,n}=\underset{i,j,m}{\sum }K{\left(S-CNN\right)}_{i,j,m,n}.\;I_{k+i-1,l+j-1,m}$$

(7) $$C_{S-CNN}=K_{H}\cdot K_{w}\cdot M\cdot N\cdot O_{H}\cdot O_{W}.$$

*D-Conv* uses a single convolution filter/kernel for each input channel or depth and *P-Conv* then applies 1 × 1 convolution to combine the outputs of the *D-Conv* and finally produce the same output as *S-CNN*. The following mathematical equation expresses the output and computational cost for *D-Conv*:

(8) $$O{\left(D-Conv\right)}_{k,l,m}=\underset{i,j}{\sum }K{\left(D-Conv\right)}_{i,j,m}.\;I_{k+i-1,l+j-1,m}$$

(9) $$C_{D-Conv}=K_{H}\cdot K_{w}\cdot M\cdot O_{H}\cdot O_{W}$$

The computational cost, *C_P-Conv_* of *P-Conv* can be expressed by

(10) $$C_{P-Conv}=M\cdot N\cdot O_{H}\cdot O_{W}$$

So the total computational cost of *DS-Conv*, *C_DS-Conv_* is

(11) $$C_{DS-Conv}=K_{H}\cdot K_{w}\cdot M\cdot O_{H}\cdot O_{W}+M\cdot N\cdot O_{H}\cdot O_{W}$$

Thus, the comparison of the reduction rate between *DS-Conv* and *S-CNN* can be calculated as follows:

(12) $$\frac{C_{DS-Conv}}{C_{S-CNN}}=\frac{K_{H}\cdot K_{w}\cdot M\cdot O_{H}\cdot O_{W}+M\cdot N\cdot O_{H}\cdot O_{W}}{K_{H}\cdot K_{w}\cdot M\cdot N\cdot O_{H}\cdot O_{W}}\;=\frac{1}{N}+\frac{1}{K_{H}K_{W}}$$

Each *DS-Conv* block comprises a *D-Conv* layer with kernels of the size of 3 × 3, and rectified linear unit (ReLU) transfer function, batch normalization (BN) layer, and *P-Conv* layer with kernels of the size of 1 × 1. Every *D-Conv* and *P-Conv* is followed by BN and ReLU. The dense block is formed as a trio of operations: dense layer, BN layer, and ReLU activation. The dense layer is a global layer where every layer is involved and connected in the following layers to all other nodes. It also allows the model to establish a global relationship among features, thereby avoiding more complex data patterns. A dropout layer is placed between dense blocks and Global Average Pooling (GAP) to prevent overfitting. The summary of the proposed HHI-AttentionNet model is presented in Table 2. 

#### 5.2.2. Antenna-Frame-Subcarrier Attention Mechanism (AFSAM)

When objects or humans move between the transmitting and receiving antennas, the moving body affects the multipath propagation, and different moves have dissimilar effects. Therefore, CSI can easily detect the information of different movements in the surrounding environment. In addition, because of the impact of multipath propagation, each subcarrier contains different information associated with human activities and the surrounding environment. Moreover, some subcarriers might be more affected by human activity, while others might be sensitive to the environment and vice versa. Furthermore, the difficulty of capturing the differences and correlations among different subcarriers concerning different frames/times makes it even more challenging to identify actual human activity data. Accordingly, the inter-antenna, inter-frame, and inter-subcarrier relationships should be used to yield different weight distributions. As a result, we proposed an antenna–frame–subcarrier attention mechanism (AFSAM) to get suitable discriminative features for various activities regardless of the surrounding environment.

##### Antenna Attention Module (AAM)

We designed an antenna attention module (*AAM*) that works based on different features’ inter transmitting-receiving antenna relationship. It mainly focuses on what are essential features and eliminates unnecessary features by refining the feature map among the transmitters-receivers. To compute the *AAM*, first we perform global average pooling to the input features *F* ϵ ℝ^*F* × *S* × *A*^, where A is the total number of antennas, *F* and *S* indicate the frame and subcarrier, respectively, and generate output $F_{gap}^{R}$.We reshape the $F_{gap}^{R}$ into *Fr* ϵ ℝ ^1 × 1 × *A*^. After that, we perform the convolution operation and apply the sigmoid activation function to get the inter-receiver attention feature map AAM(F). Then, an element-wise multiplication is performed between *AAM* output and *F*. Mathematically *AAM* can be expressed as:

(13) $$\begin{array}{c} AAM\left(F\right)=f^{1\times 1}\left(\left[Fr\right]\right)\\ =\sigma \left(f^{1\times 1}\left(\left[Fr\right]\right)\right)\;\;\;\;\;\;\; \end{array}$$

The pseudocode for the *AAM* is given in Algorithm 1.
**Algorithm 1:** The Pseudocode for the Antenna Attention Module (*AAM*)**Input**: The input feature map, *F* ϵ ℝ *^F^*
^× *S* × *A*^1: Begin2:  *F_gap_* ← ∅3:  *F_gap_* ←Globalaveragepooling (*F*)4:  *Fr*←reshape(*F_gap_*)  //After reshape operation, the input feature map, *Fr* ϵ ℝ ^1 × 1 × *A*^5:  Initialize the filter: filter^1^, filrer^2^,…, filter^n^6:  *antenna_feature* ← ∅7:  **for** *f FilterSize* **do**8:    *i* ← 09:    *temp* ←∅10:    **while** *i* ≠ filter^n^11:      *conv_i_* ←Convolute(*Fr*, *FilterSize*, padding = ‘same’)12:      append(*temp*, *conv_i_*)13:      *i*←*i* + 114:    **end while**15:    append (*antenna*_*feature*,*temp*)16:  **end for**17:  *AAM* ← Apply (*antenna_feature*, sigmoid)18: return (**F⊗**
*AAM*)19: end

##### Frame-Subcarrier Attention Module

We designed a frame-subcarrier attention module (*FSAM*) that produces spatial features by utilizing the relationship of different features between frame and subcarrier. In contrast to the *AAM*, the *FSAM* emphasizes “where”, the location of the most informative features in the spatial domain. To compute the *FSAM*, we first apply average pooling to the input features *F* ϵ ℝ *^F^_AAM_*^×*S*^*_AAM_*^×*A*^*_AAM_*, where A is the total number of antennas, *F* and *S* indicate the frame and subcarrier, respectively, and generate output $\;F_{avg}^{F_{AAM}\times S_{AAM}\times 1}$. After that, we perform a single convolution with a filter size of 5 × 5. Finally, we obtain a final *FSAM* features map by applying the sigmoid activation function on the convolution operation. Again, an element-wise multiplication is performed between the *FSAM* output and *F*. Mathematically, *FSAM* can be expressed as:

(14) $$\begin{array}{c} FSAM\left(X\right)=\sigma (f^{5\times 5}\left(\left[AvgPool\left(X\right)\right]\right)\\ =\sigma \left(f^{5\times 5}\left(\left[F_{avg}\right]\right)\right)\;\;\;\;\;\;\; \end{array}$$

The pseudocode for the *FSAM* is given in Algorithm 2.
**Algorithm 2:** The Pseudocode Frame-Subcarrier Attention Module (*FSAM*).**Input**: The input feature map, *F* ϵ ℝ *^F^_AAM_*
^× *S*^*_AAM_*
^× *A*^*_AAM_* **Output**: The frame-subcarrier attention features map1: Begin2:  *F_avg_* ←AveragePooling (*F*)3:  *frame_sub_feature* ← ∅4:  **for** *f FilterSize* **do**5:    *i* ← 06:    *temp*←∅7:    **while** *i*≠ filter8:      *conv_i_* ←Convolute(*F_avg_*, *FilterSize*, padding = ‘same’)9:      append(*temp*, *conv_i_*)10:      *i←i +* 111:    **end while**12:    append (*frame_sub_feature, temp*)13:  **end for**14:  *FSAM* ←apply(*frame_sub_feature*, sigmoid)15:  return (F**⊗**
*FSAM*)16: end

### 5.3. Hyper-Parameters and Training

Any statistical classification model comprises three steps: (i) model development phase, which requires the selection of hyperparameters, (ii) model training and validation, and (iii) model evaluation. How well a model is built and trained relies on the quantity of data with an adequate variation and selection of the proper hyperparameters such as the number of iterations, batch size, activation function, learning rate, etc. The training set is used for hyperparameter selection of the model, whereas the validation set is used for performance evaluation. The following hyperparameters were adopted for training: learning rate = 1 × $10^{-3}$, epochs = 100, batch size = 128. Additionally, a callback monitor was employed to update the learning rate. The learning rate is updated by 75% of its prior values if no improvement is seen for ten consecutive epochs. Data shuffling was allowed for training that involved shuffling the data before each epoch. The values of these hyper-parameters were selected on a trial and error basis, which provided maximum accuracy. 

Our work uses the publicly available CSI-based HHI [43] dataset to evaluate our proposed model’s performance. This dataset has no separate training and testing set. Therefore, instead of using a specific train-test split, we used the 10-fold cross-validation (CV) [46] technique to evaluate the performance of our proposed model. The 10-fold CV technique randomly partitions the entire dataset into ten non-overlapping sub-sets of equal size. It fits the models by employing an iterative procedure with nine folds, with the remaining fold being excluded for performance measurement (test and train transfer on each iteration). The overall performance in terms of recognition was determined by taking the average of the results from each iteration.

We used the Adam optimizer [47] to update weights and the cross-entropy loss function [48,49] to calculate the error/loss. The detailed procedure of class prediction and training loss computation is described in Algorithm 3.
**Algorithm 3:** Pseudocode of class Prediction and Training Loss Computation**Input:** Number of activity classes L, Dataset {(*x*_1_, *y*_1_), (*x*_2_, *y*_2_), …, (*x_n_*, *y_n_*)}, feature extractor, $f_{\varphi }$**Output:** Predicted class label $\hat{y}$, model loss J1: Randomly divide dataset into K disjoint equal-sized fold2: **For** m in 1: K **do**    loss, J = 0//Initialize loss3:    **For** *batch_size* in training set **do**4:       **For** class in classes {1… L} **do**5:         $\hat{x}=f_{\varphi }$(*batch_size*; model parameter) ϵ ℝ D (D is the dimension)6:         *α*_ij_ = Softmax (*e_ij_*) = $\frac{\exp\left(e_{ij}\right)}{\sum_{k=1}^{T_{x}}e_{ik}}$
7:        $\hat{y}\;=\hat{\;x}$. *α_ij_*//Predicated label

8:       **end for**
9:       calculate cross-entropy, J (*x_i_*, *y_i_*) = $-\overset{L}{\underset{i=1}{\sum }}y_{i}.\log{\left(\hat{y}\right)}_{i}$10:       loss = reduce_mean (J (*x_i_, y_i_*))11:       J = J $+\frac{1}{K}\log\_softmax\left(loss\right)$//loss update

12:   **end for**

13: **end for**

### 5.4. Evaluation Metrics

The performance of the proposed HHI-AttentionNet model is evaluated on the popular four performance metrics. One of them is the accuracy that reveals the model’s performance, which indicates how many predictions the model can accurately identify from the total predictions of the given dataset. However, accuracy is insufficient to show the model’s efficiency if the datasets are not balanced. As a result, we also consider the other three metrics: *F*1*-score*, Cohen’s kappa (k-score), and Matthews correlation coefficient (*MCC*). These metrics are expressed mathematically in terms of the true-positive (*TP*: the actual inspection indicates true facts, and experiments also identify true facts), the false-positive (*FP*: the actual inspection indicates false facts, and experiments also identify false facts), the true-negative (*TN*: the actual inspection indicates true facts, but experiments identify false facts), and the false-negative (*FN*: the actual inspection indicates false facts, but experiments identify true facts).

(15) $$Accuracy=\frac{TP+TN}{TP+FP+TN+FN}$$

(16) $$F1-score=2\times \frac{Precision\times Recall}{Precision+Recall}$$

(17) $$MCC=\frac{TP\times TN-FP\times FN}{\sqrt{\left(TP+FP\right)\times \left(FN+TP\right)\times \left(FP+TN\right)\times \left(TN+FN\right)}}$$

where

(18) $$Recall=\frac{TP}{TP+FN}$$

(19) $$Precision=\frac{TP}{TP+FP}$$

*Precision* defines the number of predicted true facts from total actual true facts. *Recall* identifies how frequently a model correctly detected from the true positive rate. *F*1*-score* is known as the weighted mean of recall and precision. It is more beneficial than accuracy when the dataset is uneven. It combines recall and precision for the calculation. Cohen’s kappa (k-score) tells us how well the classifier is performing compared to the performance of a classifier that randomly estimates the frequency of each class. Its value lies between 0 to 1. Matthews correlation coefficient (*MCC*) is another helpful performance metric that is not affected by imbalance in datasets and is used to calculate the differences between real and predicted values. Its value ranges from +1 to −1.

## 6. Result and Discussion

This work provides the results for the two experiments that apply the proposed HHI-AttentionNet on the CSI-based HHI dataset. We have found from the literature that some authors [41,42] have considered steady-state (no activity) as a separate class while some authors [23,38] have ignored steady-state, performed different experiments, and demonstrated the accuracy of their proposed model. Inspired by both of them, we have performed two sets of experiments (with steady-state [13 class] and without steady-state [12 class]) to demonstrate the effectiveness of our proposed HHI-AttentionNet model. Table 3 represents the resulting performance of the proposed model on the CSI-based HHI dataset for classes 12 and 13, respectively, using the 10-fold CV technique. As we can see from Table 3, our proposed model achieves an average accuracy of 94.55%, an *F*1*-score* of 94.50%, k-score of 0.945%, and *MCC* of 0.945%, for 12 classes. Our proposed model achieves an average accuracy of 95.47%, *F*1*-score* of 95.45%, k-score of 0.951%, and *MCC* of 0.95%, for 13 classes, which is the best performing result for the recognition of HHIs to date [41,42,50]. The close observation from Table 3, shows that the 10th fold achieves the highest performance for 12 classes and the 6th fold achieves the highest performance for 13 classes among 10 fold.

A close observation of the performance of the proposed models from Table 3 shows that our proposed model comparatively achieved better results for 13 classes. Two possible reasons might be mentioned. Firstly, steady-state signal patterns are very similar; the proposed model can detect them accurately and shows better accuracy. Secondly, adding a steady-state increased the total number of data samples, and the proposed model learns more perfectly, which may boost the accuracy.

**Figure 4:**
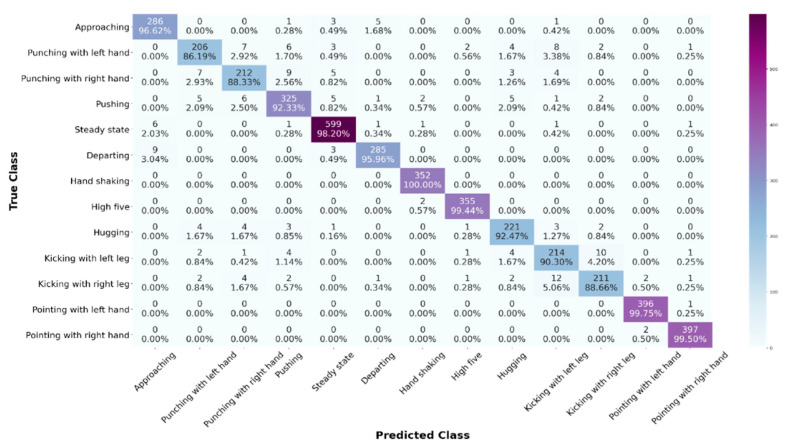
The confusion matrix of the HHI-AttentionNet model for HHIs recognition.

Figure 4 shows the confusion matrix of the proposed model, where the main diagonal represents the average recognition accuracy. Thus, all activities achieved more than 86% accuracy for 13 classes. According to the confusion matrix, our proposed model accurately recognizes pointing with handshaking interaction with 100% accuracy, although there were some mis-classification errors in other interactions. There are two main reasons for the mis-classification taking place. First, some HHI signal structures are relatively quite similar to one another, and second, the beginning and finish of some interactions are identical to steady-state interaction. We can see from Figure 4 that the maximum confusion arises from the interaction between kicking with the left leg and kicking with the right leg interaction. Similarly, the interaction between punching with the left hand and punching with the right hand has also occurred some confusion.

The number of parameters and time complexity are important factors for a deep learning model should one desire to apply it to real-world problems. Building a time-efficient model without sacrificing model performance is challenging in deep neural networks. Table 4 reports the total number of parameters, training time, and recognition time of all the considered models. Our proposed model has about 1.7 million parameters, and takes on average 3000 s seconds for training and validation. It also takes on average 0.000200 s (time in average and standard deviation values) to evaluate a single HHI. Furthermore, the proposed model uses *DS-Conv* that decreases computational cost and model size compared to other CNNs [45]. Thus, the proposed model performs better than all selected models in terms of parameters, training, validation, and recognition time.

**Figure 5:**
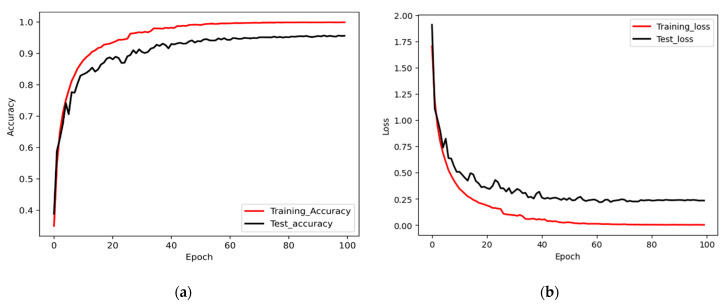
(**a**) Accuracy graph for training and testing; (**b**) Loss graph of the proposed method for training and testing.

The accuracy and loss history of our proposed model over training epochs on the training and validation sets on the CSI-based HHI dataset are shown in Figure 5. It is observed from Figure 5 that the training of the proposed model converges very rapidly within 45 epochs.

To improve the interpretability and clarity of our proposed system, we have reduced the number of dimensions of the feature representation both before and after mapping the embedding space to two dimensions, and we have visualized the results by utilizing the T-SNE algorithm.

**Figure 6:**
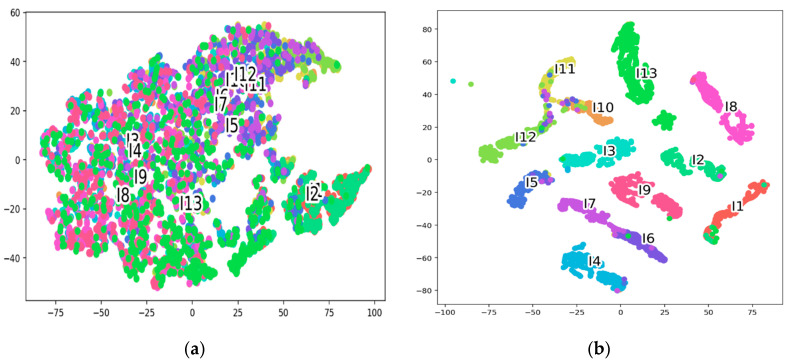
T-SNE visualization of test data before (**a**) and after (**b**) the proposed model learning representations.

We can see from Figure 6, that after the process, the distributions of features are quite different and the samples or features that belong to the same class are clustered together, whereas, before the process, the samples were congested and more challenging to identify intuitively from each other. It indicates that the proposed HHI-AttentionNet model has a highly generalized capability.

When different models are not evaluated using the same dataset, making direct comparisons between them is extremely challenging and not rational, because the performance of a model might vary depending on the dataset used for training and the quality of test samples utilized to evaluate the model’s overall performance. Therefore, we have used the same dataset, the CSI-based HHI dataset, to compare the robust performance of our proposed model with the different existing models. The performance comparison results are tabulated in Table 5. Our proposed HHI-AttentionNet model has shown higher performance than any existing work regarding HHI recognition from CSI signal compared to existing work. 

Authors [50] proposed a method to recognize HHIs from the CSI-based HHI dataset [43]. At first, they extracted eleven statistical features from the time domain and six features from the frequency domain. After this, they fed the total extracted features into the SVM classifier and achieved an overall recognition accuracy of 69.79%. On the other hand, authors [41] proposed an E2EDLF to recognize HHIs using the same dataset. They first converted the raw CSI signal into the 2-D gray image, then extracted time-domain and spatial-domain features, and finally used CNN to classify HHIs using those extracted features. Their proposed model shows an overall accuracy, and F1 score of 86.30% and 86%, respectively. However, E2EDLF requires 9.3 M trainable parameters and 0.00022 s to recognize each HHIs. Moreover, authors [41] designed a DL-based CSI-IANet model and they directly fed CSI signals to recognize HHIs after denoising. They claimed an average recognition accuracy of 91.30% and F1 score of 93%. Although CSI-IANet requires total of 4 M trainable and non-trainable parameters, which is less than E2EDLF, its recognition time is more (0.00036 s) than E2EDLF. It can be observed (Table 5) that our model displays a greater classification accuracy by over 4% compared to the existing best CNN models, retaining the same number of classes. It can be observed (Table 5) that our model displays a greater classification accuracy with about 9% better performance than E2EDLF [40] and 4% better than the CSI-IANet model [42], retaining the same number of classes. We also compared the number of trainable parameters and recognition time. It also demonstrated that our proposed model used 1.7 M trainable parameters which was either 5 times and 3 times less than the compared methods. Performance analysis thus shows that our model is more suitable than any other existing model in HHI.

## 7. Conclusions

We have proposed a lightweight DL model (HHI-AttentionNet) for automatic recognition of HHIs. Existing CNN models have been proposed for recognition of HHIs, but most of them suffer from limited recognition accuracy, require many parameters, and have high computational costs. HHI-AttentionNet uses the *DS-Conv* block as the key module to build the network, which helps to reduce the model parameters and computational costs. The combination of the *DS-Conv* block and the AFSAM increases the model’s ability to focus on the most significant features, ignoring the irrelevant features and reducing the impact of the complexity on the CSI signal; the accuracy of the proposed model improved. The performance of the HHI-AttentionNet was evaluated on the CSI HHI dataset. The experimental result shows that the HHI-AttentionNet model achieved an average accuracy of 95.47%, which is more than 4% higher than the accuracy of the existing best model. The comparisons demonstrated that the HHI-AttentionNet model is better than state-of-the-art CNN-based methods in terms of accuracy, the number of parameters, and recognition time. 

In the future, we would like to extend the work proposed in this study to recognize HHIs performed by more than two individuals in a real environment. In that case, data annotation is a tedious and complex task. Adapting semi-supervised learning [51] could be a good solution in this regard which could be the future research direction. 

## Figures and Tables

**Table 1:** Details of the CSI-based HHI dataset.

Interaction	Label	No. of Samples	Interaction	Label	No. of Samples
Approaching	I1	3359	Pointing with the left hand	I8	4067
Departing	I2	3115	Pointing with the right hand	I9	4081
Handshaking	I3	3606	Punching with the left hand	I10	2497
High five	I4	3643	Punching with the right hand	I11	2500
Hugging	I5	2480	Pushing	I12	3610
Kicking with the left leg	I6	2471	Steady state	I13	22,792
Kicking with the right leg	I7	2489			

**Table 2:** Summary of the HHI-AttentionNet model.

Section	Layer Type	Output Shape	Parameters
$\mathrm{Feature}\;\mathrm{extractor},\;f_{\varphi }$	Conv 2D	256 × 15 × 32	1760
	BN and ReLU	256 × 15 × 32	128
	*DS-Conv* block	128 × 8 × 64	2816
	AFSAM	128 × 8 × 64	4145
	*DS-Conv* block	64 × 4 × 128	9728
	*DS-Conv* block	32 × 2 × 128	18,816
	AFSAM	32 × 2 × 128	16,433
	*DS-Conv* block	16 × 1 × 256	35,840
	*DS-Conv* block	8 × 1 × 256	70,400
Recognition	GAP	1 × 256	0
	Dropout (0.20)	1 × 256	0
	Dense	1 × 64	16,448
	Softmax	1 × 13	845

**Table 3:** Performance result of the proposed model on the CSI-based HHI dataset with 10-fold CV. All results are in percentages (%).

Number of Class	Metrics (%)	Fold										Average
		1st	2nd	3rd	4th	5th	6th	7th	8th	9th	10th	
12	*Accuracy*	94.60	94.74	95.00	94.85	94.44	94.60	94.56	94.26	94.23	95.04	94.55 ± 0.25
	*F*1 *Score*	94.56	94.70	94.95	94.75	94.42	94.56	94.52	94.15	94.20	94.81	94.50 ± 0.24
	k-score	0.945	0.947	0.948	0.946	0.944	0.945	0.945	0.941	0.942	0.948	0.945 ± 0.22
	*MCC*	0.944	0.946	0.948	0.945	0.943	0.944	0.954	0.941	0.941	0.947	0.945 ± 0.38
13	*Accuracy*	95.44	95.58	95.23	95.51	95.23	95.77	95.53	95.67	95.18	95.60	95.47 ± 0.19
	*F*1 *Score*	95.41	95.56	95.21	95.49	95.22	95.74	95.51	95.66	95.16	95.55	95.45 ± 0.19
	k-score	0.950	0.951	0.948	0.951	0.947	0.954	0.951	0.953	0.947	0.953	0.951 ± 0.20
	*MCC*	0.950	0.951	0.948	0.951	0.948	0.953	0.951	0.952	0.946	0.952	0.950 ± 0.20

**Table 4:** Parameters and times of the proposed HHI-AttentionNet model.

Model	No. of Class	No. of Parameter			Time (s)	
		Trainable	Non-Trainable	Total	Training	Recognition
HHI-AttentionNet	12	173,406	2944	176,350	1615 ± 1.9	0.000198 ± 0.000012
	13	173,551	2944	176,495	3000 ± 1.4	0.000200 ± 0.000014

**Table 5:** Performance comparison of the proposed method with the existing methods on the CSI HHI dataset. Boldface denotes the highest performance, (-) denotes non-available information.

Study	Methodology and Year	Metrics (%)				Trainable Parameters	Recognition Time(s)
		*Accuracy*	F1*-Score*	k-Score	*MCC*		
Alazrai et al. [50]	SVM (2021)	69.79	-	-	-	-	-
Alazrai et al. [41]	E2EDLF (2020)	86.30	86.00	85.00	-	935,053	0.00022 ± 0.000018
Kabir et al. [42]	CSI-IANet (2021)	91.30	91.27	89.42	-	546,321	0.00036 ± 0.000025
**Proposed**	**HHI-AttentionNet**	**95.47**	**95.45**	**95.05**	**95.06**	**176,495**	**0.000200** ± **0.000014**

## Data Availability

The dataset can be found at the following website: https://data.mendeley.com/datasets/3dhn4xnjxw/draft?a=90c726d4-5493-4efc-9ee6-973bcd922b31 (accessed on 14 July 2022).
